# Electrokinetics of CO_2_ Reduction in Imidazole Medium Using RuO_2_.SnO_2_-Immobilized Glassy Carbon Electrode

**DOI:** 10.3390/molecules30030575

**Published:** 2025-01-27

**Authors:** Mostafizur Rahaman, Md. Fahamidul Islam, Zannatul Mumtarin Moushumy, Md Mosaraf Hossain, Md. Nurnobi Islam, Mahmudul Hasan, Mohammad Atiqur Rahman, Nahida Akter Tanjila, Mohammad A. Hasnat

**Affiliations:** 1 Department of Chemistry, College of Science, King Saud University, P.O. Box 2455, Riyadh 11451, Saudi Arabia; mrahaman@ksu.edu.sa; 2 Electrochemistry & Catalysis Research Laboratory (ECRL), Department of Chemistry, School of Physical Sciences, Shahjalal University of Science and Technology, Sylhet 3114, Bangladesh; 3 Department of Chemistry, Faculty of Science, Noakhali Science and Technology University, Noakhali 3814, Bangladesh; 4 Department of Applied Chemistry and Biochemistry, Graduate School of Science and Technology, Kumamoto University, 2-39-1 Kurokami, Chuo, Kumamoto 860-8555, Japan; 5 International Research Organization for Advanced Science and Technology (IROAST), Kumamoto University, Kumamoto 860-8555, Japan; 6 Department of Basic Sciences and Humanities, University of Asia pacific, Dhaka 1205, Bangladesh

**Keywords:** electrochemical CO_2_ reduction, catalysis, Tafel analysis, kinetics, convolution, renewable energy

## Abstract

The pursuit of electrochemical carbon dioxide reduction reaction (CO_2_RR) as a means of energy generation and mitigation of global warming is of considerable interest. In this study, a novel RuO_2_-incorporated SnO_2_-fabricated glassy carbon electrode (GCE) with a Nafion binder was used for the electrochemical reduction of CO_2_ in an aqueous alkaline imidazole medium. The electrode fabrication process involved the drop-casting method, where RuO_2_.SnO_2_ was incorporated onto the surface of the GCE. Electrochemical studies demonstrated that the GCE-RuO_2_.SnO_2_ electrode facilitated CO_2_ reduction at −0.58 V vs. the reversible hydrogen electrode (RHE) via a diffusion-controlled pathway with the transfer of two electrons. Importantly, the first electron transfer step was identified as the rate-determining step (RDS). A Tafel slope of 144 mV dec^−1^ confirmed the association of two-electron transfer kinetics with CO_2_RR. Moreover, the standard rate constant (*k_o_*) and formal potential (*E°*′) were evaluated as 2.89 × 10^−5^ cm s^−1^ and 0.0998 V vs. RHE, respectively. Kinetic investigations also reveal that the deprotonation and electron release steps took place simultaneously in the CO_2_RR. Based on the reported results, the GCE-RuO_2_.SnO_2_ electrode could be a promising candidate for CO_2_ reduction, applicable in renewable energy generation.

## 1. Introduction

The cumulative rise of CO_2_ in the atmosphere has been identified as one of the key drivers of the greenhouse effect and global warming [1,2,3]. Due to widespread reliance on fossil fuels, atmospheric CO_2_ levels are rising, leading to elevated global temperatures. Alarmingly, CO_2_ accounts for 76% of the total greenhouse gas emissions [3]. Consequently, researchers are actively seeking ways to mitigate CO_2_ levels by exploring its use as a renewable energy source and developing technologies for its capture and storage [4]. By converting CO_2_ into useful fuels and utilizing it as a chemical feedstock, the electrochemical or photoelectrochemical reduction of CO_2_ may provide a desirable answer to this climate-related challenge.

In recent decades, diverse techniques, such as electrochemical, photochemical, adsorption, and photoelectrochemical methods, have been applied to convert CO_2_ into valuable chemicals [5]. These techniques for CO_2_ mitigation offer distinct advantages when viewed from various perspectives. Among these techniques, the electrochemical CO_2_ reduction reaction (CO_2_RR) exhibits considerable potential for energy conversion due to its applicability to large-scale electricity generation and feasibility under ambient temperature and pressure [6,7]. Furthermore, electrocatalysts provide a greater number of active sites, a high surface area, and high porosity, all of which significantly enhance the CO_2_RR performance [8]. Given these benefits, the electrochemical reduction of CO_2_ into high-value-added chemicals offers a practically feasible approach, characterized by its simple and rapid operational process. Electrochemical CO_2_RR involves the reduction of CO_2_ to generate CO, CH_3_OH, HCOO^−^, HCOOH, HCHO, C_2_H_4_, and other value-added chemicals [9,10,11,12,13,14,15,16,17,18]. Especially, the conversion of CO_2_ into non-toxic liquid formate holds significant appeal since the conversion has substantial market value and reduces the electricity production costs [19]. Additionally, it offers potential applications in direct fuel cells as efficient hydrogen carrier systems [20,21]. Therefore, the focus of ongoing global research lies in developing durable electrocatalysts capable of selectively converting CO_2_ into formate, overcoming the considerable energy barriers associated with the CO_2_RR [22].

Notably, CO_2_ is a highly stable chemical whose reduction needs a significant amount of energy [23,24]. The low efficiency and poor selectivity of the reaction are the biggest obstacles in CO_2_RR [25]. Also, the generated products frequently combine, which are difficult to separate, resulting in low efficiency. To overcome these obstacles, researchers have investigated a variety of approaches, such as the tailoring of catalysts, customization of cell designs, and selection of solution parameters [26,27,28,29]. Pertinently, catalysts play a crucial role in CO_2_RR because they can boost the reaction rate and selectivity. Earlier investigations have revealed that transition and p-block metal electrodes, such as Au, Ag, Cu, Pd, Pt, In, Sn, Bi, Hg, and Pb, are fascinating candidates for electrochemical CO_2_ reduction reactions [30,31,32,33]. Among them, Au is the most active electrocatalyst for the reduction of CO_2_ to CO. Nevertheless, the high activation energy of CO_2_ rupture still restricts the electrocatalytic activity of Au during reduction [34]. Cu is the only metal catalyst that has been reported to generate considerable C1–C3 hydrocarbon products [35]. Sn is cost-effective with no toxicity like Bi- and Pb-based materials. Furthermore, oxides of Sn provide oxygen vacancy, grain boundaries, and low coordinated, facile sites that absorb CO_2_ and accelerate the transfer of electrons to form formic acid [36]. For instance, Kayan et al. noticed a significantly greater CO_2_ reduction capability in tin/tin oxide electrodes compared to tin foil [37]. Rende et al. also studied the CO_2_ reduction reaction with Sn/SnO_2_ that showed a Faradic efficiency of 74.7% for formate production [38]. Meanwhile, the oxides of Ru provide a lower free energy pathway as well as a high oxygen vacancy to adsorb CO_2_ [39]. Remarkably, dopants like Cu and Ru have the capability to increase the oxygen vacancy in SnO_2_ [40]. Peng et al. studied CO_2_RR with Cu- and Sn-deposited nitrogen-doped carbon cloth electrocatalysts that generated formate with 90.24% Faradic efficiency and 15.56 mA cm^−2^ current density at −0.97 V against the reversible hydrogen electrode (RHE) [41]. Similarly, other bimetallic and/or bimetallic oxide catalysts could be tailored to attain selective CO_2_RR to formate. For example, the Ru–Ru bridge sites aid in lowering the overpotential for the formation of formate, according to research by Atrak et al. that used density functional theory to assess the CO_2_ reduction reaction on TiO_2_/RuO_2_ alloy [42]. Consequently, there is a pressing need to design a bimetallic catalyst based on Sn and Ru that will display facile activation of CO_2_ and its subsequent conversion to harmless as well as profitable reduction products.

An additional noteworthy obstacle in the CO_2_ reduction process is the difficulty in amassing a reaction medium with an adequate amount of CO_2_ gas. In this context, amine compounds have been recognized for their efficacy in adsorbing CO_2_ at room temperature and ambient pressure [43]. In an aqueous environment, imidazole (C_3_N_2_H_4_) is capable of capturing CO_2_ gas and delivering a suitable amount of CO_2_ to the surface of the electrode for reduction. Additionally, amino groups in imidazole undergo protonation, leading to the formation of >NH_2_^+^ groups in the aqueous environment. These >NH_2_^+^ groups act as Lewis acids and exhibit a strong interaction with CO_2_, which acts as a Lewis base. This interaction enhances the solubility of CO_2_ [44]. The presence of active >NH_2_^+^ sites in the structure of imidazole enables improved catalytic performance during CO_2_ reduction [45].

Thus, this study aims to design a RuO_2_-incorporated SnO_2_ catalyst fabricated over glassy carbon electrode (GCE) surfaces with the assistance of Nafion. The catalytic performance and kinetics of the CO_2_ reduction reaction have been investigated. To the best of our knowledge, no such kinetic investigation pertaining to the electrochemical reduction reaction of CO_2_ was reported in any previous literature using a RuO_2_.SnO_2_ catalyst.

## 2. Results and Discussion

### 2.1. Surface Characterization

**Figure 1:**
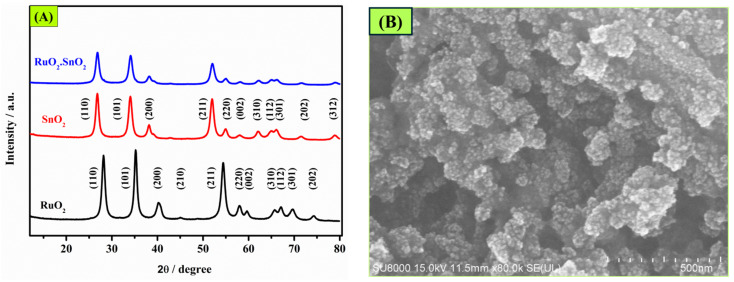

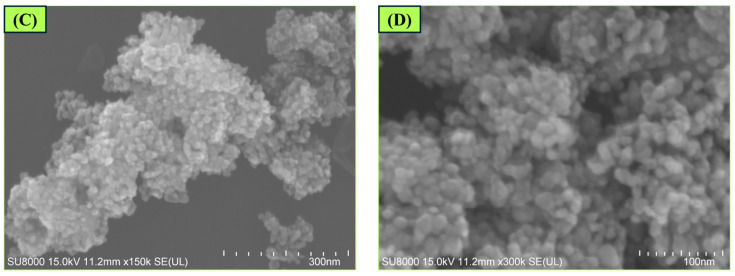
(**A**) XRD patterns of pristine RuO_2_ (black line)_,_ pristine SnO_2_ (red line)_,_ and RuO_2_.SnO_2_ composite (blue line); (**B**–**D**) SEM images of RuO_2_.SnO_2_ surface at 500 nm, 300 nm, and 100 nm magnification.

The phase compositions of the synthesized catalyst were analyzed with the help of an X-ray diffraction (XRD) study; the observed XRD pattern is presented in Figure 1A. It is seen that the crystalline phase of the pure SnO_2_ is the tetragonal rutile phase, which is also the major bulk phase found in the synthesized RuO_2_.SnO_2_ catalyst. However, peaks belonging to RuO_2_ were not found in the case of the composite catalyst, suggesting a probable formation of a solid solution through the insertion of Ru^4+^ cations into the crystal lattice of SnO_2_ [46,47,48]. The structural feature of both the oxides is almost identical, that is, tetrahedral, and both of the metallic ions have similar ionic radii, which could make the oxides prone to developing a solid solution with a defined lattice capacity [46,47,48]. Given the usage of a small amount of RuO_2_ in the synthesis process, it is conjectured that the lattice capacity of Ru is too small to detect the Ru–Sn–O solid solution by means of XRD analysis [46,47,48]. Consequently, all of the ruthenium-related species could be incorporated into the SnO_2_ lattice matrix.

In order to assess the surface morphology of the synthesized catalyst, the scanning electron microscopy technique was employed, and the resultant images are illustrated in Figure 1C,D. It is apparent from the images that the surface of the catalyst is porous and has sponge-like morphology with significant roughness and complexity. Furthermore, SEM images reveal that the particles of the synthesized catalyst are of irregular shape due to the formation of clusters of small crystals. This observation indicates substantial enhancement of surface area, which might be beneficial for electrochemical application. The enhancement of surface area as a result of the incorporation of RuO_2_ into SnO_2_ matrix was proved by means of Brunauer–Emmett–Teller (BET) and Barrett–Joyner–Halenda (BJH) techniques, as described in our previous study [49].

**Figure 2:**
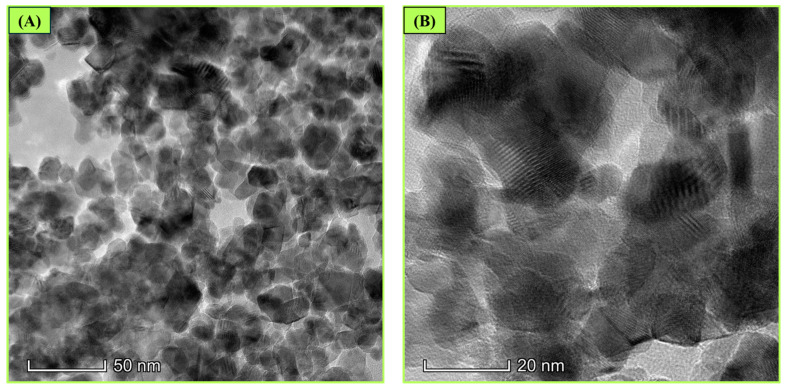
TEM images of RuO_2_.SnO_2_ surface at different magnifications: 50 nm (**A**) and 20 nm (**B**).

The transmission emission microscopic (TEM) analysis was then carried out to extract the information about the distribution of RuO_2_ particles on SnO_2_ phases. TEM images of pure SnO_2_ particles, as illustrated in Figure S1A–D in the Supporting Information file, obtained at various magnifications, demonstrate that the structures are interconnected with each other. In addition, the crystalline nature of the pure SnO_2_ particles is evident from the lattice fringes found in the high-resolution TEM images. On the other hand, RuO_2_.SnO_2_ retains a sphere-shaped structural feature similar to the pure SnO_2_ particles, but the surface of the composite is rougher compared to the SnO_2_, as presented in Figure 2A,B. 

**Figure 3:**
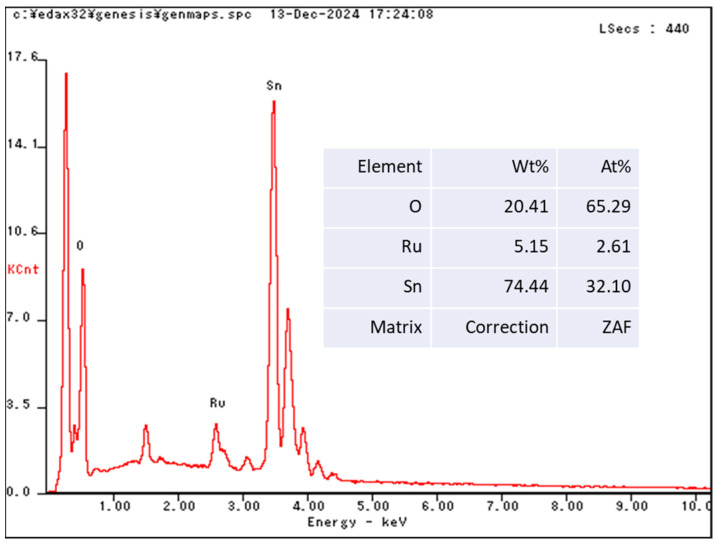
EDX spectrum of the synthesized RuO_2_.SnO_2_.

The elemental composition of RuO_2_.SnO_2_ was then determined by performing the energy-dispersive X-ray (EDX), and the resultant plot is illustrated in Figure 3. From the spectrum, it is seen that Ru, Sn, and O elements are present with a composition of 5.15 wt%, 74.44 wt %, and 20.41 wt %, respectively. These findings validate the successful formation of the RuO_2_.SnO_2_ composite. Furthermore, EDX mapping was also carried out to analyze the spatial distribution of elements, as portrayed in Figure S2. It is clearly seen that the Ru element was almost homogeneously distributed on the SnO_2_ matrix.

**Figure 4:**
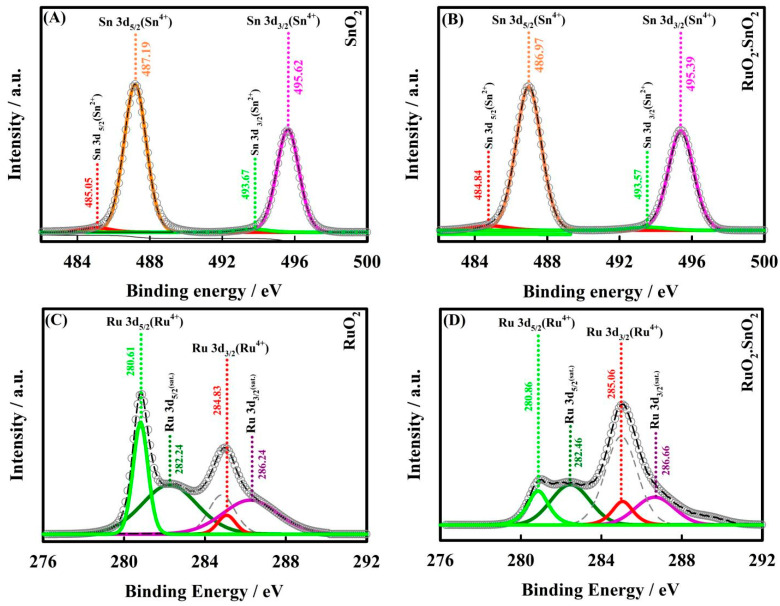

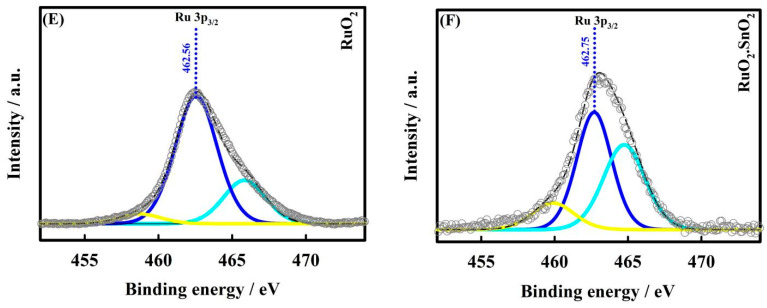
XPS spectra of Sn 3d of SnO_2_ and RuO_2_.SnO_2_ (**A**,**B**), Ru 3d of RuO_2_ and RuO_2_.SnO_2_ (**C**,**D**); and Ru 3p of RuO_2_ and RuO_2_.SnO_2_ (**E**,**F**).

At this point, the X-ray photoelectron spectroscopic (XPS) experiments were performed to investigate the surface properties of the RuO_2_.SnO_2_ catalyst. Figure 4A,B illustrates the XPS spectra of Sn 3d of pure SnO_2_ and the synthesized RuO_2_.SnO_2_, respectively. The peaks belonging to the binding energy of 487.19 eV and 495.62 eV in Figure 4A might be attributed to the existence of Sn 3d_5/2_ and Sn 3d_3/2_ of Sn(IV) species in pure SnO_2_ particles, respectively [50]. In the case of RuO_2_.SnO_2_, these two peak maxima shifted to relatively lower binding energies, appearing at 486.97 and 495.39 eV for Sn 3d_5/2_ and Sn 3d_3/2_ (see Figure 4B and Table 1), respectively. A similar shift to low binding was also noticed in the case of the peaks that correspond to the Sn^2+^ cation, as shown in Figure 4A,B and Table 1. This lower shifting of the binding energy indicates the mobilization of electrons from the Ru to Sn species while the Sn–Ru–O solid solution was formed [49,50]. The phenomenon of electron transfer between Ru and Sn was further validated from the XPS spectra of Ru 3d and 3p. The XPS analysis of pristine RuO_2_ showed the appearance of peaks in the region of 280–290 eV, as presented in Figure 4C, in which the peaks at 280.61 and 284.83 eV after deconvolution can be attributed to Ru 3d_5/2_ and Ru 3d_3/2_ of Ru^4+^ cations, respectively [51,52]. These two peaks of Ru^4+^ ion in the case of RuO_2_.SnO_2_ shifted to the higher binding energy, as illustrated in Figure 4D, confirming the electronic transition from Ru to Sn metallic species. The shifting of peaks to higher binding energy was also observed in the case of Ru 3p, which is evident from the comparison of the peak positions of Ru 3p in RuO_2_ and RuO_2_.SnO_2_, as depicted in Figure 4E,F. A comparison of the major XPS peaks for Sn and Ru is presented in Table 1 for a better understanding in support of the spectra in Figure 4. Since the composite is formed by the combination of two oxide materials, it is necessary to examine the surface oxygen properties of the catalysts. In this regard, the XPS spectra of O 1s of pristine SnO_2_ and RuO_2_.SnO_2_ were analyzed.

**Figure 5:**
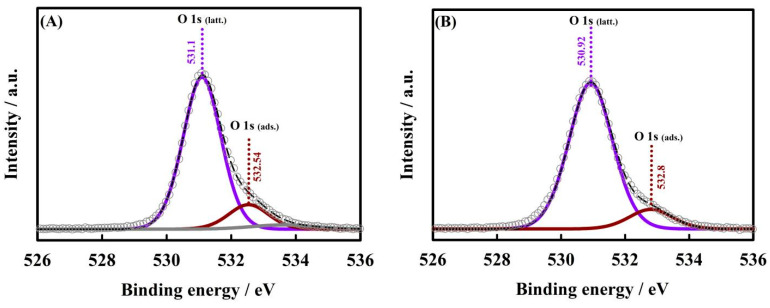
XPS spectrum of O 1s of (**A**) SnO_2_ and (**B**) RuO_2_.SnO_2_.

From Figure 5A,B, it is apparent that two peaks at 531 eV and 532.5 eV are present in both catalysts, and these peaks can be attributed to lattice oxygen (O_lat_) and adsorbed oxygen (O_ads_), respectively [50]. The molar ratio of these two different types of oxygen, that is, O_ads_/O_lat_, in the case of both SnO_2_ and RuO_2_.SnO_2_ was evaluated to be ca. 0.132 and 0.174, respectively. The increased molar ratio in the case of composite RuO_2_.SnO_2_, however, supports the development of the Sn–Ru–O bond in the solid-state material. Almost similar findings were also reported in our previous work, where this composite was synthesized for HER application [49]. However, due to the formation of solid solutions, lattice distortion may result in defect formation, which may enhance the generation of surface mobile oxygen species [53,54,55].

### 2.2. Electrochemical Characterization

**Figure 6:**
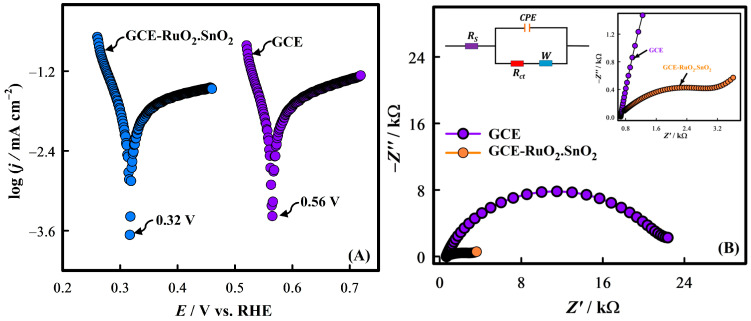
(**A**) Linear polarization at GCE and GCE-RuO_2_.SnO_2_ in 0.05 M imidazole, (**B**) EIS spectra of pristine GCE and GCE-RuO_2_.SnO_2_ at −0.58 V vs. RHE recorded with CO_2_ in N_2_-saturated 0.05 M Imidazole solution; inset shows the equivalent circuit and magnified part. Inset of Figure 6B: *R_s_* = solution resistance; *R_ct_* = charge transfer resistance; *W* = Warburg element; *CPE* = constant phase element.

The electrochemical characterization of the GCE-RuO_2_.SnO_2_ electrode was accomplished through linear polarization and electrochemical impedance spectroscopy (EIS) measurements. Open circuit potential (OCP) was determined by linear polarization to characterize the electrode material where the potential at zero current is measured. Figure 6A shows the polarization curves for bare GCE and GCE-RuO_2_.SnO_2_ in 0.05 M imidazole. As the GCE-RuO_2_.SnO_2_ electrode was expected to be catalytically viable, the nature of the electronic charge developed was assessed when the GCE-RuO_2_.SnO_2_ interface interacted with CO_2_ at open circuit conditions in 0.05 M imidazole. The OCP value examined with a bare GCE was found to appear at 0.56 V vs. RHE. Conversely, when a GCE-modified RuO_2_.SnO_2_ electrode was employed, the OCP appeared relatively at a more negative potential (0.32 V vs. RHE) in reference to a bare GCE. This observation confirms that the fabricated GCE-RuO_2_.SnO_2_ electrode acquired additional negative charges i.e., a more reducing environment on its surface, indicating a provable enhancement in the adsorption process of CO_2_.

To perceive the provable charge transfer properties of GCE and GCE-RuO_2_.SnO_2_, the EIS was recorded in CO_2_-saturated imidazole by applying a potential below the OCP value, e.g., −0.58 V vs. RHE, as an excitation potential. Figure 6B demonstrates the typical appearance of Nyquist plots at bare GCE and GCE-RuO_2_.SnO_2_ electrodes in the presence of CO_2_ in 0.05 M imidazole. In EIS analysis, the size of the semicircle observed in the Nyquist plot corresponds to the charge transfer resistance (*R_ct_*) of the electrode surface, and a higher *R_ct_* value suggests that the reaction kinetics are slower [56,57,58]. In this research, the GCE-RuO_2_.SnO_2_ composite unveiled a smaller semicircle diameter with an *R_ct_* of 3.19 kΩ, while at bare GCE, the *R_ct_* value was observed to be 20.9 kΩ. The lower polarization resistance at the GCE-RuO_2_.SnO_2_ electrode compared to a bare GCE indicates that the CO_2_RR reduction activity is more convenient at GCE-RuO_2_.SnO_2_ in comparison to a bare GCE, since this decrease in polarizable character indicates the formation of catalytic sites at the electrode surface. The equivalent circuit is shown as the inset of Figure 6B, while the relative EIS parameters of the electrode processes are reported in Table 2.

### 2.3. Cyclic Voltammetry

**Figure 7:**
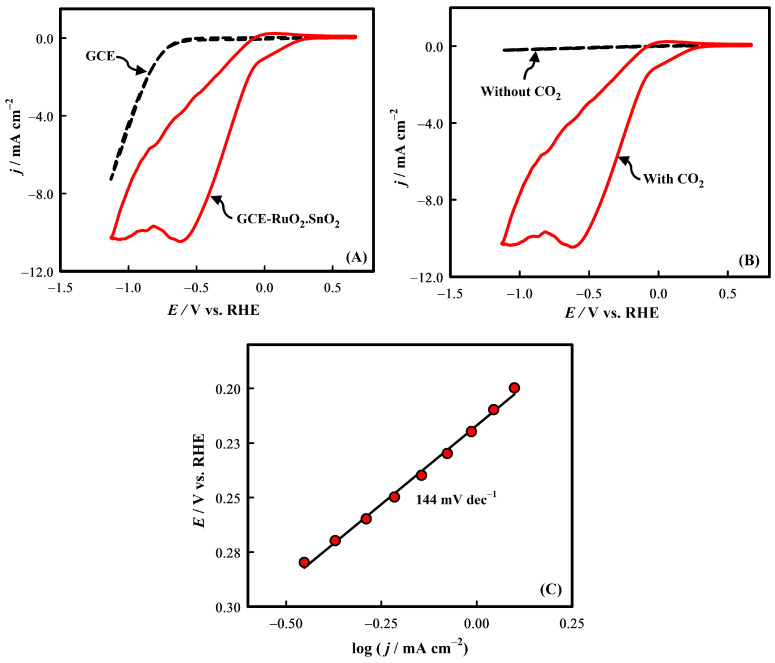
CVs of (**A**) bare GCE (Black dash) and GCE-RuO_2_.SnO_2_ (Red) with CO_2_, (**B**) CVs of GCE-RuO_2_.SnO_2_ with and without CO_2_, and (**C**) Tafel plot of GCE-RuO_2_.SnO_2_ electrode in 0.05 M imidazole at 0.1 Vs^−1^ scan rate.

As outlined in the previous section, the EIS investigation revealed that the GCE-RuO_2_.SnO_2_ electrode potentially produces catalytic sites for CO_2_ reduction. Consequently, to assess the catalytic performance, CV analysis was conducted at both the GCE and GCE-RuO_2_.SnO_2_ electrodes using a CO_2_-saturated imidazole solution employing a 0.1 V s^−1^ scan rate. Figure 7A shows diffusive currents resulting from the reduction of CO_2_ with a sharp peak at −0.58 V vs. RHE at the GCE-RuO_2_.SnO_2_ electrode, while bare GCE exhibits no peak in 0.05 M imidazole solution in the presence of CO_2_. The peak at −0.58 V vs. RHE for the reduction of CO_2_ further clarified when the reaction was carried out at the GC-RuO_2_.SnO_2_ surface between the potential regions of 0.67 V and −1.13 V vs. RHE with and without CO_2_, as shown in Figure 7B. Note that the GCE modified with RuO_2_.SnO_2_ showed no peak in the absence of CO_2_ but while the medium was saturated with CO_2_, a sharp peak appeared, which confirms the reduction of CO_2_ at the electrode surface. By contrast, at the pristine GCE electrode, a potential-dependent kinetic current was observed. This comparable observation implies that under the experimental conditions, the GCE-RuO_2_.SnO_2_ electrode possesses more active catalytic sites than a pristine GCE electrode to execute CO_2_ reduction. Briefly, when RuO_2_.SnO_2_ nanocomposites are immobilized on the GCE surface, a robust catalytic effect is generated pertinent to a quicker electron transfer rate with an onset potential (*E_i_*) of 0.26 V vs. RHE and a peak potential (*E_p_*) of −0.58 V vs. RHE. It is worthwhile to note that no significant competition from the HER was observed in the working potential range as depicted by the dashed-line CV obtained without CO_2_ (blank) in the N_2_-saturated imidazole solution in Figure 7B. Furthermore, we have achieved one of the highest current densities as well as lower peak potentials in a well-defined diffusive CV, indicating that the electrode’s behavior is activation-controlled for CO_2_RR and is favorable to investigating peak-related kinetics [59]. While numerous studies have been conducted on the electrochemical CO_2_ reduction, only a few have managed to achieve a diffusive nature of the CV in CO_2_ reduction [60]. Table 3 provides a comparison between various electrochemical CO_2_RR parameters reported in our findings and several previously reported studies.

### 2.4. Kinetics

#### 2.4.1. Tafel Analysis

The Tafel analysis is a valuable tool for studying the electrochemical process because it provides insights into reaction kinetics, mechanism, and catalyst performance. By analyzing the Tafel slope under various experimental conditions, it is possible to gain a deeper understanding of the CO_2_ reduction pathways and develop more efficient and selective processes for converting CO_2_ into valuable products (such as carbon monoxide, formate, or methane). Therefore, to delve deeper into the electrochemical CO_2_RR pathway, Tafel analysis was performed at the kinetic domain of the voltammogram using the following Equation (1) [68,69,70]:

(1) $$\log\left(j\right)=\log(j_{k})+b_{T}(E-{E{}^{\circ}}^{\prime })$$

Herein, $\left(E-{E{}^{\circ}}^{\prime }\right)$ represents overpotential, and $b_{T}$ is the Tafel slope. The value of $b_{T}$ was calculated as 144 mV dec^−1^ from the correlation of log(*j*) and *E* vs. RHE, as shown in Figure 7C. Numerous research groups have previously analyzed the Tafel slopes of CO_2_RR to determine the reaction pathway. The Tafel slope in this study (144 mV dec^−1^) is consistent with the mechanism of CO_2_ reduction to formate, which involves the addition of one electron to the adsorbed CO_2_ to generate carbon dioxide radical anion as the initial RDS (rate-determining step), according to earlier studies [37,68,71,72,73]. The findings reveal that CO_2_RR at the GCE-RuO_2_.SnO_2_ electrode surface involves the ${2e}^{-}$ transfer mechanism producing formate, where the conversion of CO_2_ to CO radical anion is the RDS.

#### 2.4.2. Scan Rate Effect

**Figure 8:**
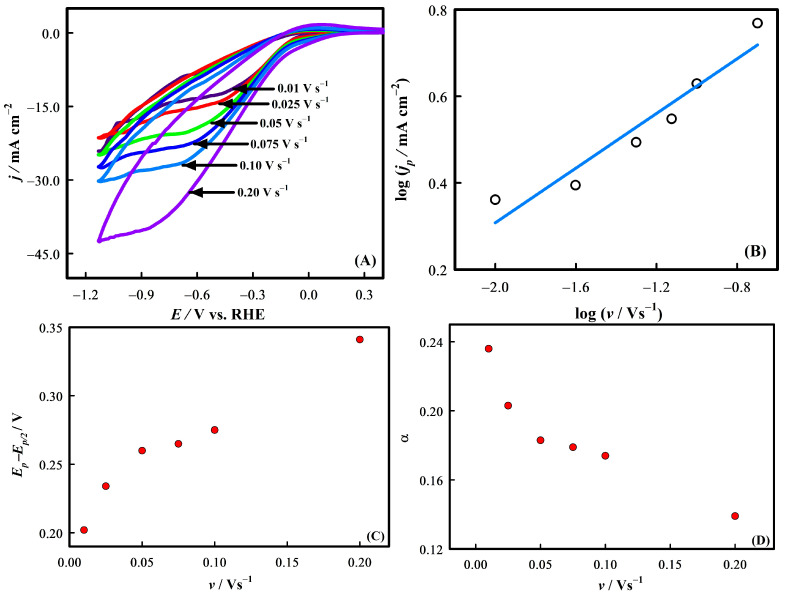
(**A**) CVs of saturated CO_2_ in 0.05 M Imidazole over GCE-RuO_2_.SnO_2_ electrode at various scan rates, (**B**) log *j_p_* vs. log *v*, (**C**) *E_p_–E_P/2_* vs. *v*, and (**D**) α vs. *v* plot.

Determination of the kinetic parameters of electron transport (ET) relies significantly on the scan rate. To uncover the kinetic parameters of CO_2_RR at the GCE-RuO_2_.SnO_2_ electrode, CVs were recorded at various scan rates ranging from 0.010 to 0.2 V s^−1^ (Figure 8A). It is seen that the CO_2_RR current increased with the increase in scan rate; afterward, the corresponding slope (log *j_p_* vs. log *υ*) was found to be 0.32 (Figure 8B), which suggests that the CO_2_RR at the GCE-RuO_2_.SnO_2_ electrode follows mass transfer (diffusion-controlled) kinetics [68]. The above phenomenon is also correlated to the underlying character of the diffusion-controlled irreversible charge transfer process. This result indicates that the electron transfer step is striking upon the adsorption step on the surface at potentials more negative than the onset potential (0.0998 V vs. RHE). The typical characteristics of the CVs regarding *E_i_*, *E_p_*, and *j_p_* observed due to the increased scan rate ensure that the GCE-RuO_2_.SnO_2_ electrode is stable and reproducible for CO_2_RR.

Nonetheless, the transfer coefficient (α) distinguishes between the various categories of ET routes concerned in an irreversible reaction. Equations (2) and (3) show how α of an irreversible process is correlated to the activation-free energy (∆*G*^‡^(*E*)) and reaction-free energy (∆*G^o^*) [68]. According to the formulas, if α varies linearly with potential (*E*), the ET kinetics could be described by the Butler–Volmer (B–V) model:

(2) $$\alpha =\frac{{∆G}^{‡}(E\;)}{{∆G}^{o}}=\frac{{∆G}^{‡}(E\;)}{F(E-E^{o})}$$

(3) $$\alpha =\frac{{∆G}^{‡}(E)}{{∆G}^{o}}=0.5+\frac{F(E-E^{o}))}{{{∆G}^{‡}}_{o}}$$

where ∆*G*^‡^*_o_* represents the activation-free energy while *E* = *E^o^*. A theoretical analysis of the B–V kinetics suggests that the peak potential (*E_p_*) increases linearly with the logarithm of the scan rate (υ). Furthermore, α is related to both the peak potential and half-peak width potential (*E_p_*_/2_) at 298 K, according to the following Equations (4) and (5) [68]:

(4) $$\frac{{\partial E\;}_{p}}{\partial log(v)}=\frac{29.6}{\alpha }\;mV$$

(5) $$E_{p}-E_{\frac{p}{2}}=\frac{47.7}{\alpha }mV$$

The dependence of (*E*-−*E_P_*_/2_) on the scan rate is plotted to determine the reaction pathway as shown in Figure 8C. When the scan rate was increased from 0.010 to 0.20 V s^−1^, a significant variation in both E_p_–E_P/2_ (shifted from 0.202 to 0.341 V) and the corresponding α (altered from 0.24 to 0.14) was observed (see Figure 8C,D). This observed variation suggests that the B–V method is not suitable for explaining the ET kinetics of the CO_2_RR at the GCE-RuO_2_.SnO_2_ surface [68]. It is worthwhile to note that the B–V model is only applicable to the kinetic region of the voltammogram.

#### 2.4.3. Convolution Study

The Butler–Volmer (B–V) model is restricted in its applicability to the kinetic region and requires the transfer coefficient (α) to remain constant within this range. The earlier scan-rate-dependent analysis reveals that the transfer coefficient of CO_2_RR on GCE-RuO_2_.SnO_2_ is not invariant and changes with the scan rate. In light of this limitation, Convolution Potential Sweep Voltammetry (CPSV) emerges as a more robust and precise method for determining the transfer coefficient (α). Unlike the B–V model, CPSV enables accurate estimations not only within the kinetic region but also across the entire voltammogram, ensuring a comprehensive analysis. The convolution of voltammetric current generates a classic sigmoid curve including a plateau. According to Equation (6), the peak corresponds to the limiting convolution current (*I_l_*) as follows [68,74,75]:

*I*_*l*_ = *nFAC√D*_*o*_ (6)

Here, *n* is the number of electron transfers, *F* is Faraday’s constant, *A* stands for the surface area of the electrocatalyst, *C* represents the concentration of the electroactive species, and *D_o_* is the diffusion coefficient.

**Scheme 1:**
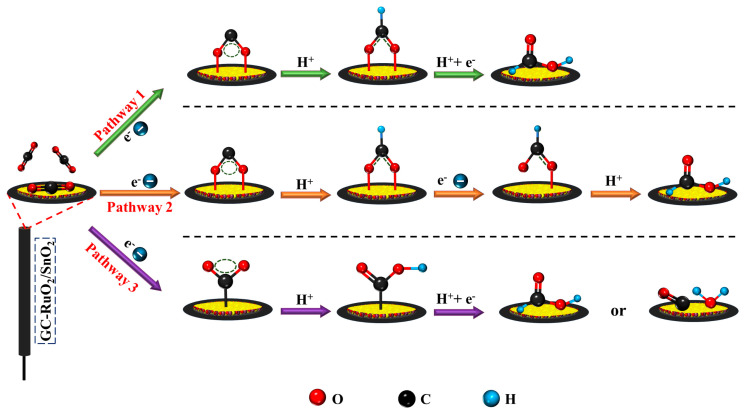
Provable CO_2_ reduction pathways on GCE-RuO_2_.SnO_2_ electrocatalytic surface. Replicated from [77] and accessible under a CC-BY 4.0 license. Copyright 2019, Zhao et al.

This limiting current is self-reliant on scan rate and can be employed to calculate ‘*n*’ or ‘*D_o_*’ almost precisely if the capacitive current is correctly extracted. As per a previous report, the diffusion coefficient (*D_o_*) of carbon dioxide is 1.71 × 10^−5^ cm^2^ s^−1^ [76]. By applying this value of *D_o_*, the number of electrons involved in CO_2_RR was calculated to be 1.92 (≈2) using Equation (6). The proposed reaction pathways corresponding to a 2e^−^ transfer CO_2_ reduction reaction are presented as Scheme 1.

The production of formate and CO can occur via three different pathways, as depicted in Scheme 1. However, the choice of pathway largely relies on whether the initial proton coupling occurs at the carbon or oxygen atom of the adsorbed CO_2_ radical anion. If protonation takes place at the carbon atom, it forms an HCOO· intermediate, leading to pathway 1, where subsequent electron transfer and protonation result in HCOOH formation. Pathway 2 involves an additional step where HCOO^−^ is converted to the ·OCHO intermediate via electron transfer, followed by protonation for HCOOH formation. Pathway 3 initiates with protonation at the oxygen atom, forming a ·COOH intermediate, which then undergoes electron transfer and protonation to yield either HCOOH or CO, releasing water. To unveil more on kinetics, the transfer coefficient was next evaluated by exploiting CPSV.

**Figure 9:**
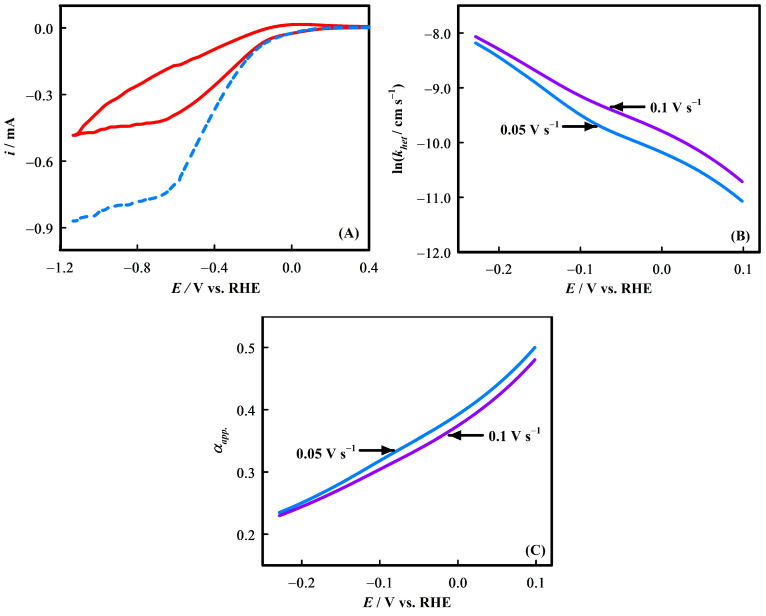
(**A**) The CV (solid line) and convoluted current (dotted line) of CO_2_ reduction in 0.05 M Imidazole at 0.1 V s^−1^ scan rate; (**B**) Plot of natural logarithmic heterogeneous rate constant (ln*k_het_*) against applied potential (*E*) at 0.05 and 0.1 V s^−1^ scan rate; and (**C**) *α_app_* vs. *E* plots at 0.05 and 0.1 V s^−1^ scan rate.

Figure 9A displays the CPSV currents measured at 0.1 V s^−1^, and using the value of the limiting current, the heterogeneous rate constant (*k_het_*) can be calculated for an irreversible ET process as per Equation (7) [68], where *I*_(*l*)_, *i*_(*t*)_, and *I*_(*t*)_ stand for the limiting convolution current, cyclic voltammetric current, and time-dependent convolution current, respectively. Applying Equation (8) within a limited potential range, the apparent transfer coefficient (α_app_) was finally calculated [68,78] as follows:

(7) $$\ln k_{het}=ln\sqrt{D}-\ln\frac{I_{\left(l\right)}-I_{\left(t\right)}}{i_{\left(t\right)}}$$

(8) $$\alpha_{app}=\frac{RT}{F}\frac{dlnk}{dE}$$

Figure 9B,C display the plots of ln*k_het_* vs. *E* and α vs. *E*, respectively, in the case of two different scan rates. The curving behavior observed in Figure 9B suggests that the ET kinetics of CO_2_RR at the GCE-RuO_2_.SnO_2_ surface was potential-dependent [68,78]. From Figure 9C, it is evident that the α value was ca. 0.5 near the onset potential (0.0998 V vs. RHE) and then declines to 0.23 around −0.2202 V vs. RHE. This decrease in α (α < 0.5) within the potential range (0.0998 V to −0.2202 V vs. RHE) suggests that the protonation and electron release steps occur simultaneously [68,72]. Furthermore, α*_app_* becomes equal to 0.5 at *E* = *E°*′, which indicates a nonlinear electron transfer process [79]. Thus, by adjusting *α_app_* = 0.5 in Figure 9C, the formal potential (*E°*′) value regarding CO_2_RR was calculated to be 0.0998 V vs. RHE, which is consistent with the OCP value. Finally, the standard rate constant (*k^o^*) was estimated as 2.89 × 10^−5^ cm s^−1^. Therefore, at this point, it can be concluded that the CO_2_RR mechanism at the GCE-RuO_2_.SnO_2_ electrode surface is highly dependent on the applied potential, where a potential of 0.0998 V vs. RHE represents the breakthrough point.

**Scheme 2:**
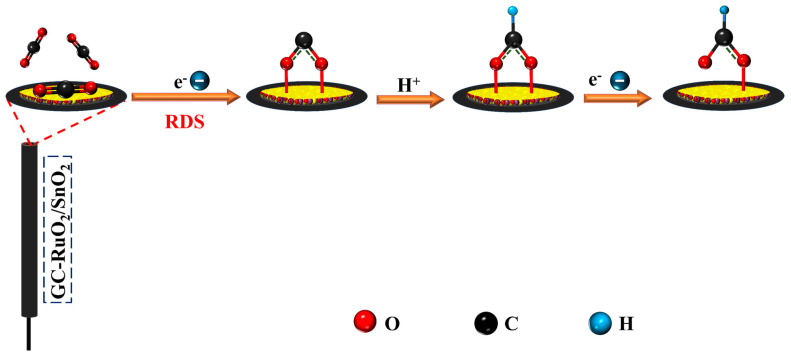
CO_2_ reduction pathway at the GCE-RuO_2_.SnO_2_ electrode surface.

An α value less than 0.5 indicates the presence of a slow step, in which radical species (COO•−) are formed following the initial ET [80]. This observation further suggests a two-step ET process, with the first step being the slowest, followed by a faster heterogeneous electron transfer. Consequently, the rate of radical anion formation on the catalytic surface is slower compared to the rate of the second ET [81]. The initial ET step, becoming the RDS in CO_2_RR over the developed GCE-RuO_2_.SnO_2_ electrode, strongly suggests the formation of formate anion via a two-electron transfer step, as illustrated in Scheme 2 [82].

**Figure 10:**
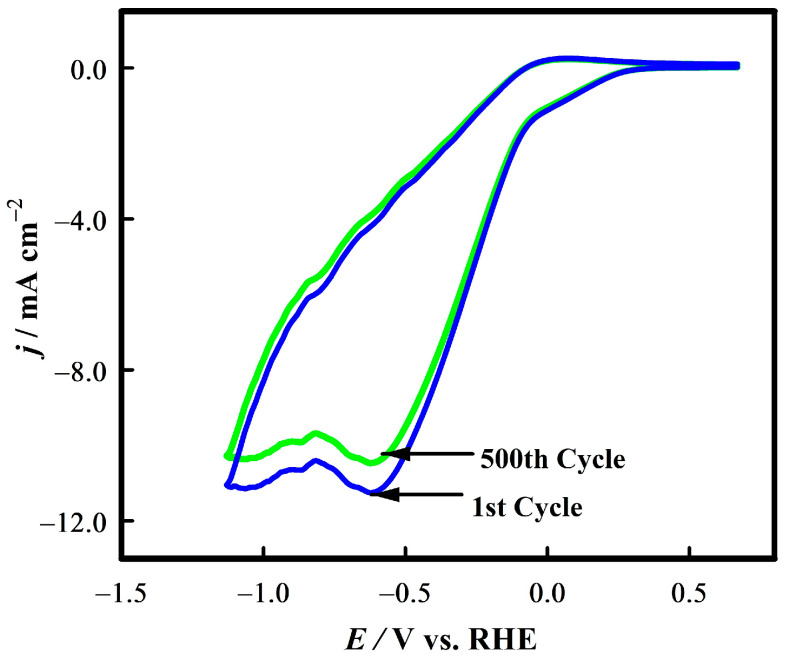
CVs of CO_2_ reduction reaction obtained using GCE-RuO_2_.SnO_2_ electrode in CO_2_-saturated 0.05 M imidazole solution for the 1st and 500th potential scanning at 0.1 V s^−1^.

The stability of the GCE-RuO_2_.SnO_2_ electrode was then investigated by conducting 500 CV cycling with the electrode in CO_2_-saturated imidazole solution. However, no significant change in the onset potential (*E_i_*), peak potential (*E_p_*), and peak current (*I_p_*) was observed after 500 cycles of CV runs, boasting an impressive stability of the proposed electrode, as shown in Figure 10.

## 3. Experimental

### 3.1. Chemicals and Instruments

All the chemicals used in this research were of analytical grade and used without further purification. The major chemicals, such as SnCl_4_ and RuCl_3_, were purchased from Wako (Japan). Meanwhile, urea was collected from Shahjalal fertilizer industry (Sylhet, Bangladesh). In all cases, solutions were prepared with ultrapure deionized water having resistance nearly 18.1 MΩcm.

The pure CO_2_ gas was obtained in high-pressure liquid form and stored in a cylinder for dissolution into an aqueous imidazole medium. It is important to note that employing high-pressure/supercritical CO_2_ rather than 99.99% pure CO_2_ gas has some benefits, such as being more affordable, leaving no unwanted compounds behind, and being conveniently easy to store [83]. Potentiostat PGSTAT 128N (Metrohm Autolab BV, Kanaalweg 29G, 3526 KM Utrecht, The Netherlands), CHI602E electrochemical workstation (CH Instruments Inc., 3700 Tennison Hill Dr, Bee Cave, TX 78738, USA), and Wavedrive 20 (Pine Research Instrumentation, Inc. 2741 Campus Walk Avenue, Building 100, Durham, NC 27705, USA) were used to perform all the electrochemical experiments in combination with the globally acknowledged three-electrode system.

### 3.2. Synthetic Method of RuO_2_.SnO_2_ Catalyst

At first, SnO_2_ was synthesized following homogeneous precipitation method. Shortly, 5 g SnCl_4_ was mixed with 20 g urea in a beaker, and then 100 mL of deionized (DI) water was added to it. The resulting mixture was then stirred at 200 rpm at 90 °C in a magnetic bath for 4 h until it started to reflux. The refluxing process was continued until a white precipitate was formed. The as-developed precipitate was then centrifuged and washed with required amount of DI water prior to drying at 120 °C. The dried material as developed was pulverized with a mortar until fine powder was obtained. The as-prepared material was heated at 500 °C for 3 h under aerated conditions in a furnace, and powdered SnO_2_ material was obtained by pulverizing again in a mortar. Next, 650 mg SnO_2_ and 65 mg RuCl_3_ were mixed in a beaker containing 50 mL of DI water. The mixture was magnetically agitated for ca. 4 h at 200 rpm. The blend was immediately exposed to heating at 160 °C until all the water was removed via evaporation. After removal of water, the resultant solid material was left in an oven at 120 °C overnight. The composite material obtained in this way was crushed to fine powder using a mortar, which again was heated at 500 °C for 3 h in a muffle furnace. Finally, a homogeneous RuO_2_.SnO_2_ composite was obtained by pulverizing the material after cooling it to room temperature.

### 3.3. Morphological and Chemical Characterization

A powder X-ray diffractometer (Rigaku D/MAX RINT-2000) operating at 40 kV and 40 mA with Cu Kα radiation was used to determine the crystal structures of the synthesized catalysts. Diffraction patterns were recorded in continuous scan mode over a 2θ range of 10° to 80°, with a sampling pitch of 0.1° and a scan rate of 2° min^−1^. X-ray photoelectron spectroscopy (XPS) was performed with Al Kα monochromatic radiation (12 keV) using a K-Alpha spectrometer (Thermo Fisher Scientific) to assess the valence states and chemical bonding characteristics. Surface charge effects were corrected using the C 1s binding energy at 285 eV as a reference, and all spectra were normalized to Al 2p for quantitative analysis.

### 3.4. Electrode Preparation

For cyclic voltammetric measurements, Nafion-stabilized RuO_2_.SnO_2_-modified glassy carbon electrode (GCE-RuO_2_.SnO_2_; geometric area of 0.07 cm^2^) has been used as the working electrode (WE). At first, the GCE was polished with 0.3 μm alumina slurries until a smooth, glossy surface was observed. After polishing, the electrode was exposed to ultrasonication in the presence of methanol and acetone for a duration of 20 min at 25 °C to remove abrasive particles from the electrode surface. Following the ultrasonic treatment, the GCE was cleaned in 0.1 M H_2_SO_4_ by maintaining the potential between −1.2 and 0.5 V at 0.1 V s^−1^ for 100 cycles until a repeatable cyclic voltammogram (CV) corresponding to the characteristic behavior of GC was achieved. After electrochemical cleaning, the GCE surface was capped with the as-developed RuO_2_.SnO_2_ catalyst following drop-casting method. To prepare catalyst ink, exactly 0.5 mg of RuO_2_.SnO_2_ catalyst was suspended in a homogeneous solution of 75 µL ethanol and 25 µL of Nafion (5 wt%), and the mixture was continuously stirred for 10 min under ultrasound conditions. Then, 5 µL of the catalyst ink (RuO_2_.SnO_2_) was pasted on the cleaned GCE disk surface and left overnight in open air, which resulted in a GCE-RuO_2_.SnO_2_ electrode. The electrode surface was finally dried for 4 h at room condition. Herein, Nafion polymer acted as a binder and a supportive layer by stabilizing the drop-casted RuO_2_.SnO_2_ catalyst over the GCE surface.

### 3.5. Electrochemical Measurements

An internationally recognized three-electrode configuration was used for the electrochemical investigations of CO_2_RR in a 0.05 M imidazole electrolyte solution. The WE was GCE-RuO_2_.SnO_2_, while Pt and Ag/AgCl (saturated in KCl) were used as the counter and reference electrode, respectively. To elucidate the electronic properties of the GCE-RuO_2_.SnO_2_ electrode, linear polarization curves were recorded in a 0.05 M imidazole solution. Next, to perceive the provable charge transfer properties of the catalyst, the EIS of the electrode was recorded in CO_2_-saturated imidazole by applying a potential below the OCP value, e.g., −0.43 V vs. RHE, as an excitation potential. To assess the catalytic performance, Cyclic voltammetric (CV) analysis was conducted with and without CO_2_-saturated imidazole solution, employing 0.1 V s^−1^ scan rate. The mass transfer effect was studied under saturated conditions of CO_2_ by altering the scan rate between 0.01 and 0.2 V s ^−1^. Convolution potential sweep voltammetry analysis was carried out using the CHI660 electrochemical workstation by subtracting the background current. Note that all potentials were adjusted to the reversible hydrogen electrode (RHE) scale with 80% iR compensation applied using the following Equation (9):

(9) $$E_{RHE}=E_{Ag/AgCl(saturated\;KCl)}+0.197+\left(0.0591\times pH\right)-iR$$

## 4. Conclusions

An RuO_2_.SnO_2_-covered GCE was used for the CO_2_ reduction reaction in an alkaline imidazole medium. From the voltammetric responses, it was deduced that the catalyst selectively produces formate at moderate overpotential. The convolution study indicated the involvement of an RDS among the two-step electron transfer kinetics that served as proof for the production of formate over CO during CO_2_ reduction at the GCE-RuO_2_.SnO_2_ electrode surface. A scan rate-dependent CV analysis uncovered that the reaction is diffusion-controlled, whereas the convolution study suggested that the deprotonation and electron release steps occur simultaneously during the CO_2_RR. Overall, these findings provide a new dimension for the use of Ru–Sn-based energy materials in CO_2_ reduction reaction, thus offering numerous valuable insights for further understanding of the CO_2_ reduction kinetics toward formate production.

## Figures and Tables

**Table 1:** XPS peak position of the metal ions in pristine SnO_2_, RuO_2_, and the synthesized RuO_2_.SnO_2_ catalysts.

Catalyst	Sn 3d_5/2_ (Sn^2+^)/eV	Sn 3d_3/2_ (Sn^2+^)/eV	Sn 3d_5/2_ (Sn^4+^)/eV	Sn 3d_3/2_ (Sn^4+^)/eV	Ru 3d_5/2_ (Ru^4+^)/eV	Ru 3d_3/2_ (Ru^4+^)/eV	Ru 3p_3/2_ (Ru^4+^)/eV
SnO_2_	485.05	493.67	487.19	495.62	-	-	-
RuO_2_	-	-	-	-	280.61	284.83	462.56
RuO_2_.SnO_2_	484.84	493.57	486.97	495.39	280.86	285.06	462.75

**Table 2:** EIS parameters of the bare GCE and GCE-RuO_2_.SnO_2_ electrodes recorded in CO_2_-saturated 0.05 M Imidazole solution.

Electrode	*R_s_* (Ω)	*R_ct_* (kΩ)	*CPE* (µMho)
GCE	669	20.9	64.9
GCE-RuO_2_.SnO_2_	790	3.19	3.36

*R_s_* = solution resistance; *R_ct_* = charge transfer resistance; *CPE* = constant phase element.

**Table 3:** Comparison of CV parameters for different electrodes regarding CO_2_RR.

Electrodes	−*E_p_*/V vs. RHE	*j*_*p*_/mA cm^−2^	*υ*/Vs^−1^	[CO_2_]/ mM	Solvent	Ref.
GCE	1.60	0.25	0.01	5	BMP TFSI	[61]
Cu	1.93		0.1	454	BMP TFSI	[62]
Ag	1.38	0.1	0.5	45	[BMIM] [TFSI]	[63]
[Co^II^(bipy)_3_](BF_4_)_2_/GCE	1.31	0.38	0.1		TBAPF6 + acetonitrile	[64]
SnS|PTFE|Pt	0.43	1.52	0.05	18	imidazole	[12]
[(Mn(TPP)Cl)]/VCE	0.73	6.0	0.1	230	acetonitrile	[65]
[Re(BPEBP)(CO)_3_Cl]/Pt	1.03	2.6	0.1	280	acetonitrile	[66]
Co(TPP)Cl/VCE	0.83	0.0176	0.1		[Bu_4_N][BF_4_]-acetonitrile + DMF	[67]
GCE-RuO_2_.SnO_2_	0.58	10.49	0.1	18	imidazole	This work

*E_p_*: peak potential; *j_p_*: peak current density; and *υ*: scan rate.

## Data Availability

The data presented in this study are available on request from the corresponding author.

## Supplementary Materials

The following supporting information can be downloaded at: https://www.mdpi.com/article/10.3390/molecules30030575/s1, Figure S1: TEM images of SnO_2_ at the magnifications; Figure S2: Elemental mapping in RuO_2_.SnO_2_.
