# Supplementary material for: Electrokinetics of CO2 Reduction in Imidazole Medium Using RuO2.SnO2-Immobilized Glassy Carbon Electrode
Source: Molecules. 2025 Jan 27;30(3):575. doi: 10.3390/molecules30030575 (PMC11820317; doi:10.3390/molecules30030575)
Supplement: Supplementary file 1 [file molecules-30-00575-s001.zip › molecules-3428298-supplementary.pdf]

Supporting Information

Electrokinetics of CO_2 reductoion in imidazole medium using $RuO_2.SnO_2$ immobilized glassy carbon electrode

Mostafizur Rahaman[a], Md. Fahamidul Islam[b, c], Zannatul Mumtarin Moushumy[d], Md Mosaraf Hossain[b], Md. Nurnobi Islam[b], Mahmudul Hasan [b], Mohammad Atiqur Rahman[d], Nahida Akter Tanjila[f] and Mohammad A. Hasnat [b,e,*]

[a]*Department of Chemistry, College of Science, King Saud University, P.O. Box 2455, Riyadh 11451, Saudi Arabia*

[b]*Electrochemistry & Catalysis Research Laboratory (ECRL), Department of Chemistry, School of Physical Sciences, Shahjalal University of Science and Technology, Sylhet-3114, Bangladesh*

[c]*Department of Chemistry, Faculty of Science, Noakhali Science and Technology University, Noakhali-3814, Bangladesh*

[d]*Department of Applied Chemistry and Biochemistry, Graduate School of Science and Technology, Kumamoto University, 2-39-1 Kurokami, Chuo, Kumamoto, 860-8555, Japan*

[e]*International Research Organization for Advanced Science and Technology (IROAST), Kumamoto University, Kumamoto 860-8555, Japan*

[f] *Department of Basic Sciences and Humanities, University of Asia pacific, Dhaka-1205, Bangladesh*

***Correspondence: mah-che@sust.edu**

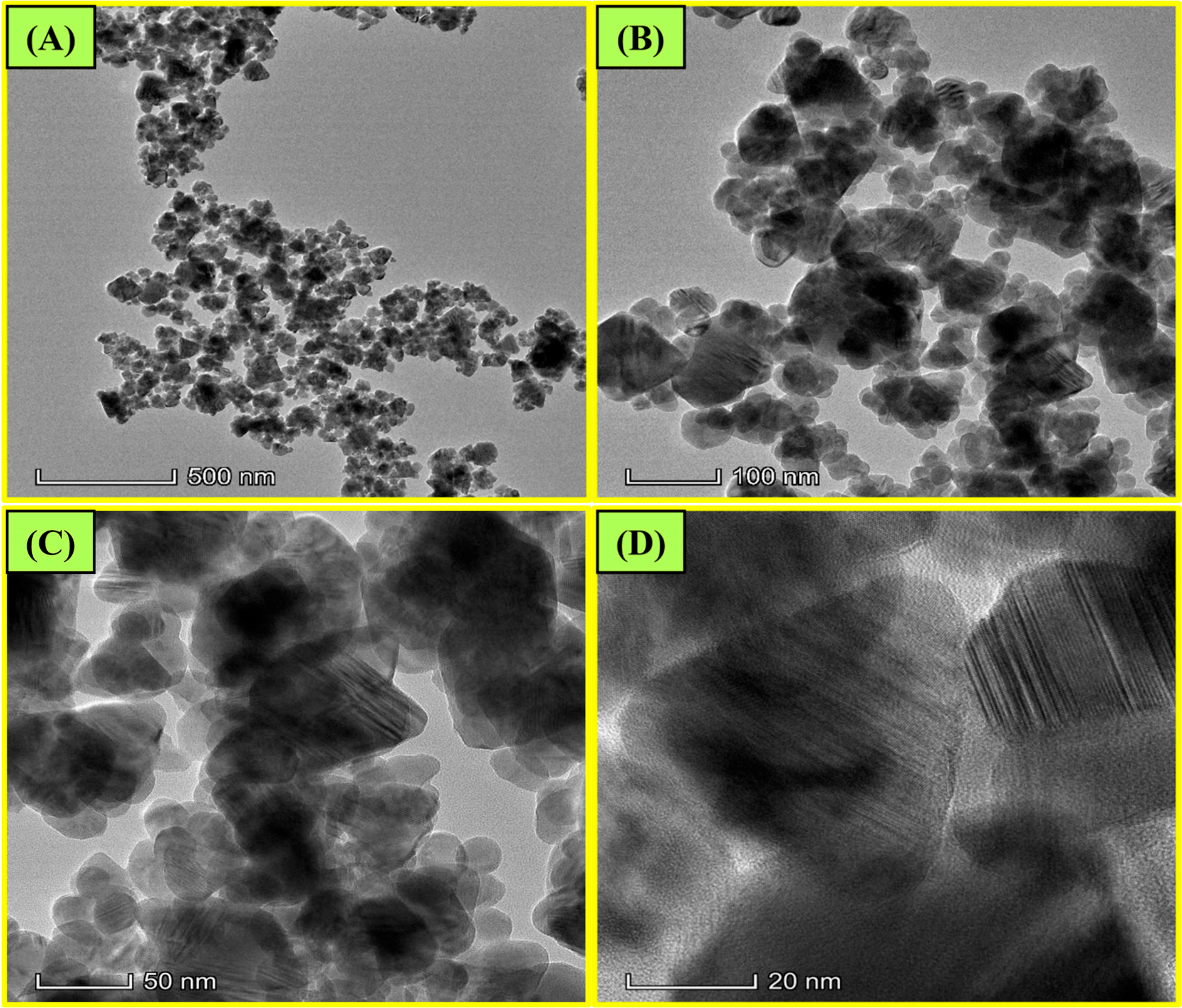

Figure S1. TEM images of of SnO_2 at the magnifications of **(A)** 500 nm, **(B)** 100 nm, **(C)** 50 nm **and (D) 20 nm**.

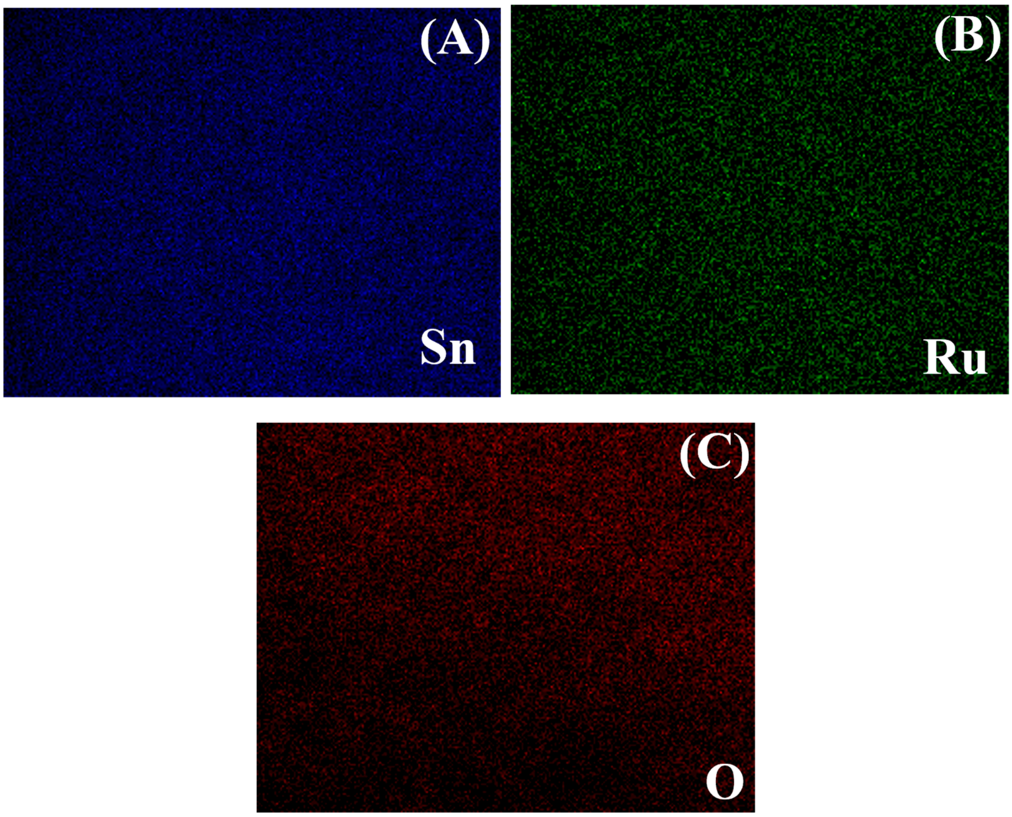

Figure S2. Elemental mapping of (A) Sn, (B) Ru and (C) O in $RuO_2.SnO_2$.
